# Improving Vietnamese Immigrants’ Cognitive Health Literacy: University-Community Partnerships

**DOI:** 10.1093/geroni/igaf122.3245

**Published:** 2025-12-31

**Authors:** Christina Miyawaki, Nguyen Nguyen, Tuong-Vi Ho, Angela McClellan

**Affiliations:** University of Houston, Houston, Texas, United States; University of Houston, Houston, Texas, United States; Texas Woman’s University, Houston, Texas, United States; University of Houston, Houston, Texas, United States

## Abstract

After the fall of Saigon in 1975, waves of Vietnamese refugees/immigrants migrated to the United States (U.S.). Currently, 2.3 million Vietnamese live in the U.S. Many came with adverse conditions; however, their health data is scarce. After 50 years, many are aged and may have unmet health needs. To fill this gap, we developed the Vietnamese Aging and Care Survey and collected their health data in Houston, Texas, which revealed a high prevalence of physical, mental, and cognitive disabilities. Using the university-community partnerships, we developed a linguistically and culturally tailored dementia one-pager, formed the Cognitive Health Initiative (CHAIN), and offered Vietnamese refugees/immigrants complimentary memory tests. This study explains how the CHAIN program operates and fosters intergenerational relationships. Using the Cultural Exchange Model, we trained bilingual/bicultural Vietnamese college students (Cohort 1) and conducted cognitive assessments, Vietnamese Montreal Cognitive Assessment (V-MoCA), in 2023. In 2024, when Cohort 2 students joined, we formed mentor (Cohort 1)-mentee (Cohort 2) dyads. Cohort 1 demonstrated the assessment while Cohort 2 observed their mentors perform assessments. After several demonstrations, Cohort 2 tried the assessments supervised by Cohort 1. They repeated this sequence until Cohort 2 felt comfortable conducting the assessments independently. During 2023-2024, we recruited 42 students, attended 24 health fairs, and offered 406 V-MoCA. Mentors (Cohort 1) completed training their mentees (Cohort 2) with tips for conducting the V-MoCA and how to work with older adults. Through the mentor-mentee relationships, the CHAIN promoted teamwork, accountability, ethics, and responsibility to the community, and demonstrated successful university-community partnerships.

